# Notes on the Methods of Performing Paracentesis of the Various Cavities

**Published:** 1894-10-13

**Authors:** John Poland

**Affiliations:** Surgeon to the Miller Hospital, Greenwich


### Medical Progress and Hospital Clinics.

[*The Editor will be glad to receive offers of co-operation and contributions from members of the profession. All letters should be addressed to* The Editor, The Lodge, Porchester Square, London, W.]

### NOTES ON THE METHODS OF PERFORMING PARACENTESIS OF THE VARIOUS CAVITIES—*(Continued)*.

By John Poland, F.R.C.S., Surgeon to the Miller Hospital, Greenwich.

**Tapping the Abdomen.**—Two years ago Dr. Cheadle, before the British Medical Association, insisted upon the great importance of early paracentesis, and I cannot, in conclusion, do better than quote his words on the subject: "I would urge then that the fluid which does so much harm should be removed early, before it causes serious pressure on the viscera. The functions of the various organs will be restored, and time gained for the development of the collateral circulation. It is certain that in many of these cases enough liver tissue remains to carry on life, if only the portal circulation can be sufficiently eased, and the passage of nutriment into the systemic vessels sufficiently established by the development of the collateral channels. This is, however, a work of time. Frequently the patient is carried off before it is effected by the fatal effect of the ascitic pressure, by engorgement of lungs, by hydrothorax, by general failure from embarrassment of organic functions. To gain time is of the essence of cure. This can only be done with promptness and certainty by paracentesis, and with an amount of wear and tear infinitely less than by purging, or drugging with diuretics in the vain attempt to produce diuresis. The operation is, I repeat, perfectly free from risk if properly performed, at any rate in the case of patients who have not reached the very last stage of exhaustion.

"Then, no doubt, tapping is sometimes followed by rapid sinking. I presume that this fact, and the not infrequent supervention of peritonitis, in former days led to the belief that after paracentesis the end would soon arrive, and to the counsel of delay. But there is no danger of sinking if the operation is done early; no risk of peritonitis if the trocar is aseptic. I have seen harm result from paracentesis three times; twice in the same week eight years ago, in the early days of antiseptic precautions, from peritonitis due to the use of the same imperfectly-purified trocar; once since from collapse in a woman utterly broken down by alcoholic excess. The great number of cases known in which tapping has been repeated without ill effect, even hundreds of times, point to the intrinsic harmlessness of the operation."

**Paracentesis of the Bladder.**—There are several methods of tapping the bladder for retention of urine from stricture of the uretha or any other cause. (1) Puncture per rectum is now almost given up on account of the danger of wounding the prostate and the difficulty of performance when this gland is enlarged, of the formation of an abscess between the rectum and bladder, and of injury to the vescialœ seminales or peritoneum; after the operation difficulty of defecation and even permanent fistula are serious drawbacks; moreover the tube often slips out and has to be reintroduced. The operation was a good advance introduced by Mr. Cock before the days of antiseptic and aseptic surgery.

(2) Puncture of the bladder through the perineum for enlarged prostate (recommended by Mr. Harrison) is also fraught with many of the same dangers as the last procedure, viz., injury to the prostate, &c.

(3) Supra pubic cystotomy is sometimes useful for habitual retention of urine, due to enlargement of the prostate after other alleviating treatment has failed.

(4) Supra pubic aspiration with a fine needle; as a temporary measure no one can doubt its utility, but it must only be employed in this manner. When one puncture gives sufficient temporary relief for the obstruction to be overcome, the bladder reached by the natural passage and the catheter passed—the plan is a good one. It is not however without some risk of leaking at the seat of puncture and the formation of an abscess, especially if the walls of the bladder are in an unhealthy condition. To relieve the pain of puncture a good plan is to press the finger firmly against the pubis.

By far the best and most permanent method is (5) supra pubic puncture for all forms of retention and persistent retention with enlarged prostate. The tube may be retained for some time, and the supposed liability to extravasation of urine is not borne out by practice. In cases of enlarged prostate the size of the prostate often diminishes, and the urine passes in the natural way. It is much easier to perform than puncture per rectum, which it has entirely superseded. It does not interfere with defecation, and the patient is able to have control over the cannula. The patient being placed in a recumbent position with the pubis shaved and cleansed, a small vertical incision is made through the skin, and subcutaneous tissue, just above the symphysis down to the linea alba, and the tip of the left fore-finger introduced. The bladder being steadied on either side by the hands of an assistant, a curved trocar with the convexity upwards is then thrust into the bladder. The trocar is then removed and the cannula tied in, and some india-rubber tubing attached to it, or to an inner tube or catheter introduced through the cannula. The puncture must be immediately above the pubis, or else the peritoneum may be wounded.

**Paracentesis Capitis,** or tapping the lateral ventricles in young children before ossification of the skull is complete. It is usually performed at the coronal suture with a fine needle in cases of chronic, not acute, hydrocephalus. Repeated punctures at intervals of a few weeks have not been followed by many cases of cure. The operation in itself is not dangerous, but the disease is a grave one.

**Tapping of the Membrana Tympani and Anterior Chamber of the Eye** for collections of pus and certain other conditions involve special considerations which need not be considered in the present paper.

